# Body mass index and risk of lung cancer: Systematic review and dose-response meta-analysis

**DOI:** 10.1038/srep16938

**Published:** 2015-11-19

**Authors:** Peng Duan, Chunhui Hu, Chao Quan, Xianfu Yi, Wei Zhou, Meng Yuan, Tingting Yu, Ansoumane Kourouma, Kedi Yang

**Affiliations:** 1 MOE (Ministry of Education) key lab of Environment and Health, Department of Occupational and Environmental Health, Tongji Medical College, Huazhong University of Science and Technology, Wuhan, Hubei, China; 2 Department of Clinical Laboratories, the Affiliated Taihe Hospital, Hubei University of Medicine, Shiyan, Hubei, China; 3 Department of Emergency, the Affiliated Taihe Hospital, Hubei University of Medicine, Shiyan, Hubei, China

## Abstract

Questions remain about the significance of the dose-response relationship between body mass index (BMI) and lung cancer (LC) risk. Pertinent studies were identified through a search in EMBASE and PUBMED from July 2014 until March 2015. The summary relative risk (SRR) and confidence interval (CI) were estimated. The dose-response relationship was assessed using a restricted cubic spline. The overall meta-analysis showed evidence of a nonlinear association between BMI and LC risk (*P*_nonlinearity_ < 0.001). The SRR were 0.98 (95%CI: 0.95–1.01) for 25 kg/m^2^, 0.91 (95%CI: 0.85–0.98) for 30 kg/m^2^ and 0.81 (95% CI: 0.72–0.91) for 35 kg/m^2^, with mild between-study heterogeneity (*I*^2^ = 5%). The results of the stratified analysis by gender were comparable to those of the overall meta-analysis. When stratified by smoking status, linear dose-response associations were observed for current smokers, ex-smokers and non-smokers (*P*_nonlinearity_ > 0.05), whereas the effects were attenuated when restricting analysis to non-smokers, and at the point of 30 kg/m^2^, the SRR was 0.96 (95%CI: 0.86–1.07) for males and 0.95 (95%CI: 0.89–1.02) for females. This meta-analysis provides quantitative evidence that increasing BMI is a protective factor against LC. Keeping normal-to-moderate BMI should be prescribed as an evidence-based lifestyle tip for LC prevention in smokers.

Lung cancer (LC) is one of the most prevalent and deadliest human cancers. The average five-year survival rate after diagnosis is 16.3%^1^, so it is essential to emphasise the importance of LC prevention and knowledge of modifiable risk factors. The identification of environmental exposures predisposing individuals to the development of LC, such as tobacco smoking and air pollution^2^,^3^,^4^,^5^,^6^, is prevalent among good quality epidemiology studies that explain the majority of LC incidence. Notably, the complex interplay of aetiological and psychophysical factors is believed to modify the effect of respiratory carcinogens on LC initiation and prognosis^7^,^8^. Growing evidence indicates that LC is one outcome for which the role of BMI requires clarification^8^,^9^,^10^, but the results from epidemiological studies are sometimes conflicting.

Several observational epidemiological studies have shown that higher BMI correlates with a lower risk of LC^6^,^7^,^8^,^11^. Two recent meta-analyses have provided more evidence supporting the idea that excess weight could significantly decrease the risk of LC^12^,^13^. It has been hypothesised that excess body weight and obesity are protective factors against LC, especially in current and former smokers. Despite this, the inverse association between BMI and LC risk is often criticised due to inadequate adjustment for cigarette smoking^11^,^14^. Some observations failed to confirm this association^9^,^15^, and have even reported contradictory results^16^. Moreover, BMI had been found to be unrelated to LC when limited to non-smokers^17^,^18^. Thus, clarifying the association between BMI and the risk of LC stratified by smoking status has important public health implications.

No work to date has clarified the dose-response relationship between BMI and LC risk, not to mention the differences relative to the classification of smoking status. In addition, the previous meta-analysis was performed simply by combining the same categories of BMI across individual studies and the resulting conclusions have been questioned. We therefore carried out a dose-response meta-analysis of prospective cohort studies to provide a more precise evaluation for the potential association between BMI and LC risk. We particularly wanted to clarify potential modification by smoking status, as well as to investigate potential heterogeneity by subgroup and meta-regression analyses.

## Methods

### Literature search and selection

A comprehensive literature retrieval was conducted by using the databases PubMed (Medline) and Embase from 6th July 2014 until 13th July 2014. An updated secondary search was performed until 1st March 2015. Pertinent articles were identified by using the key words “body mass index,” “BMI,” “obesity,” “overweight,” “body size” or “body weight” combined with “lung cancer,” “lung neoplasm” or “lung carcinoma”. Additionally, we scrutinized references from relevant original papers and review articles to obtain other pertinent publications. No language restrictions were imposed. We followed the criteria and standard for conducting meta-analyses of observational studies and reporting the results^19^.

### Inclusion and exclusion criteria

Three authors independently retrieved and assessed potentially relevant articles according to the prespecified selection criteria. Discrepancies between reviewers were solved by discussion. To be included, the study had to meet the following criteria: (a) published as an original article; (b) has definite description of BMI in kg/m^2^; (c) with cohort design; (d) investigators provided the following data: the number of cases, the total subjects or person-years and estimates of relative risk (RR) [hazard ratio (HR), rate ratio, or standardized incidence ratio] with 95% confidence intervals (95%CIs) for three or more quantitative categories of BMI. If the person-years were not reported, they were calculated through multiplying the number of subjects by mean follow-up duration^20^; if multiple articles were on the same study population, the one with complete design or larger sample size was finally selected. Additionally, when there were separate data for gender, smoking status, or lung cancer histology in one study, they were considered apart^21^.

### Data extraction

Two reviewers designed and pilot-tested an ad hoc developed excel sheet for data extraction, which was eventually approved by the authors’ team. General information, study characteristics, participants’ characteristics, results as reported (including number of participants, reference population, estimates of RR with 95%CI for BMI categories), associated raw data for re-calculation (data checking) and any multivariate analyses adjustment factors (if applicable) were collected.

For each study, the median or mean value for each category of BMI was assigned to each corresponding estimate of RR. When the median or mean value per category was not obtained in the study, we assigned the mid-point of the upper and lower boundaries in each category as the average BMI. For open-ended categories, value equal to 0.83 (1/1.2) times the upper limit of the lowest category and equal to 1.2 times the lower limit of the highest category were consistently used^22^.

### Quality assessment

To assess study quality of the included studies, a 9-point scoring system according to the Newcastle-Ottawa Scale was used^23^. Two stars with respect to comparability of studies on the basis of the design and analysis. A high-quality study was defined as a study with ≥7 points.

### Statistical analysis

In this meta-analysis, the relative risks (RRs) and 95%CIs were considered as the effect size for all studies. Because the incidence of LC is low, the HR from cohort studies is approximated to RR. Besides, we just included RR or HR based on the Cox proportional hazard model for statistical analysis. Thus we reported all risk estimate produced by current meta-analysis as RR for simplicity. We extracted the RRs from the maximally adjusted model to lower the risk of possible confounding.

We followed the WHO international classification and defined body mass categories as follows: underweight (BMI < 18.5 kg/m^2^), normal (BMI = 18.5–24.9 kg/m^2^), overweight (BMI = 25–29.9 kg/m^2^), and obesity (BMI ≥ 30 kg/m^2^). Normal group (BMI = 18.5–24.9 kg/m^2^) was considered as reference group^13^. We conducted separate meta-analysis for different levels of BMI. Summary relative risks (SRRs) and 95%CIs were calculated by using an inverse variance-weighted method^24^. Stratified analyses, if three or more studies were available per subgroup, were carried out. Heterogeneity between studies was quantitatively assessed by the Cochran Q test (P < 0.1, significant heterogeneity) and I^2^ statistic (I^2^ < 30%, no heterogeneity or marginal heterogeneity; I^2^ = 30–75%, mild heterogeneity; I^2^ > 75%, notable heterogeneity)^25^. DerSimonian and Laird random-effects models were applied when heterogeneity was significant; otherwise, a fixed-effects model was applied^26^. Further restricted maximum likelihood (REML) based random effects meta-regression analysis was conducted to investigate the sources of heterogeneity among studies according to the selected factors^27^.

A dose-response meta-analysis was performed to examine a potential nonlinear relationship between BMI and LC. We used restricted cubic splines with 3 knots at fixed 10%, 50% and 90% to model a potential curvilinear relationship and then a dose-response meta-analysis was conducted using the generalized least-squares trend command for summarised dose-response data^28^,^29^. The pooled RRs for specific BMI values were finally estimated (per 0.5 kg/m^2^ increase from BMI = 20 to 35 kg/m^2^) using the restricted maximum likelihood method in a multivariate random-effects meta-analysis^30^. A *P* value for non-linearity was computed by testing the null hypothesis that the coefficient of the second spline was equal to zero^29^. Further stratified analyses, if feasible, were performed according to sex, smoking status and histologic type, respectively. Moreover, we performed sensitivity analyses to assess whether the SRRs are robust to inclusion of studies^31^. Publication bias was evaluated with the use of the Begg and Egger’s test^32^. All statistical analyses were performed with Stata 10.0 software.

## Results

### Search results and study characteristics

The detailed steps of our literature search are shown in Figure S1. Briefly, we identified 29 cohort studies of BMI and LC risk. These studies included 56,189 cases and 7,253,941 participants. Among the studies included, 14 studies provided RRs with 95%CIs for underweight, 29 for overweight and 29 for obesity. Meanwhile, 11 studies were conducted in the United States, nine in Europe, and nine in Asia. Most studies controlled for age (27 studies) and smoking (26 studies). Nine and seven articles only reported separate outcomes of males and females, respectively, and 13 articles reported the outcomes of both sexes. Of these 13 articles, five provided data of males and females combined, while eight reported outcomes of males and females separately. Detailed characteristics of the cohort studies, including study effect estimates, are provided in Table S1. Study-specific quality scores are summarised in Table S2. Overall, six studies had a score of 9, 12 had a score of 8, eight had a score of 7, and the three remaining studies had a score of 6.

### Overall risk of LC and subgroup analysis

**Figure 1:**
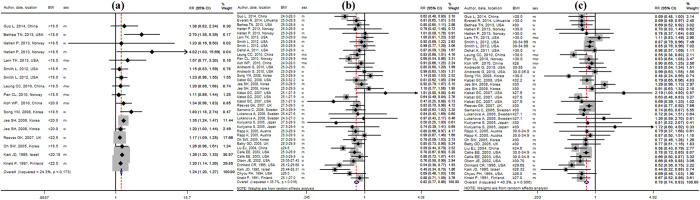
Forest plot of relative risks of underweight, overweight and obesity vs. normal weight for BMI with LC risk. Open blue diamonds denote the summary relative risks. Black diamonds indicate the RR in each study. The size of each box indicates the relative weight of each study in the meta-analysis. Horizontal lines represent the 95% confidence intervals (CIs). (**a**) Forest plots of risk of lung cancer associated with underweight (BMI ≤ 18.5 kg/m^2^). (**b**) Forest plots of risk of lung cancer associated with overweight (BMI = 25–29.9 kg/m^2^). (**c**) Forest plots of risk of lung cancer associated with obesity (BMI ≥ 30 kg/m^2^). RR, relative risk; normal weight, BMI = 18.5–24.9 kg/m^2^; BMI, body mass index; LC, lung cancer; m, men; w, women; mw, men and women.

A total of 14 studies reported an association between being underweight (BMI < 18.5 kg/m^2^) and overall LC events. The SRR showed that being underweight, compared with the reference, was associated with an increased risk of LC (RR, 1.24; 95%CI: 1.20–1.27; *P*_heterogeneity_ = 0.17; Fig. 1a). Table 1 gives pooled RRs and corresponding 95%CIs of LC risk in those that are underweight versus the reference weight in the strata of selected factors. Interestingly, being underweight was not associated with risk of LC if the individuals were ex-smokers (RR, 1.39; 95%CI: 0.82–1.27) and non-smokers (RR, 1.18; 95%CI: 0.90–1.54). Additional stratified analyses were not performed according to histological types because of the lack of data.

The pooled RRs for the association between excess weight (BMI ≥ 25 kg/m^2^) and the risk of LC were 0.82 (95%CI: 0.77–0.86) for overweight (Fig. 1b), and 0.78 (95%CI: 0.74–0.83) for obesity (Fig. 1c). A significant heterogeneity between studies was found with overweight (*P*_heterogeneity_ = 0.02) and obesity (*P*_heterogeneity_ = 0.01). Therefore, we carried out subgroup analyses to identify sources of heterogeneity and minimise heterogeneity among eligible studies. As shown in Table 2, we noted that being overweight was unrelated to the risk of LC in the small cell carcinoma group (RR, 1.04; 95%CI: 0.91–1.18). Furthermore, when the analysis was restricted to the three studies that had a NOS score of 6, the pooled RR was 1.10 (95%CI: 0.88–1.39). For the category of obesity, the results of the stratified analyses are also presented in Table 2. The association of obesity with lower LC risk was consistent in all subgroups except for the small cell carcinoma group (RR, 0.84; 95%CI: 0.56–1.26) and having the number of LC cases ≤ 200 (RR, 0.79; 95%CI: 0.58–1.08).

### Overall dose-response association between BMI and risk of LC

**Figure 2:**
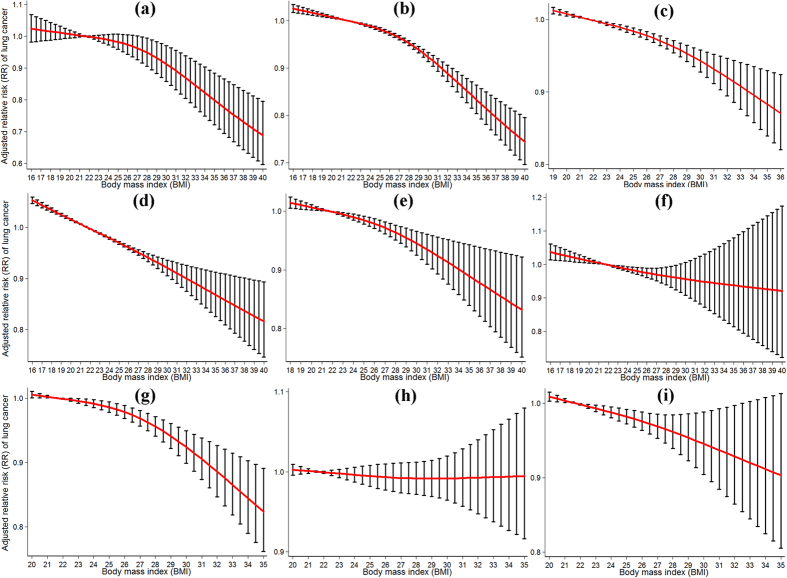
Relative risk function and the corresponding 95% confidence intervals (CIs), describing the best-fitting dose-response relationship between the BMI level and LC risk: (**a**) Overall analysis, including 26 cohort studies. (**b**) Men; (**c**) Women; (**d**) Current smokers; (**e**) Ex-smokers; (**f**) Never smoker; (**g**) Adenocarcinoma; (**h**) Small cell carcinoma; (**i**) Squamous cell carcinoma. BMI = 21.7 kg/m^2^, the mid-point of normal weight (BMI = 18.5–24.9 kg/m^2^), was served as a reference (RR = 1). The vertical lines indicate 95%CIs.

For the dose-response analysis, data from 26 studies (only high-quality studies) were used, including 43,393 LC cases. The SRR and 95%CI of LC per five-unit increment in BMI were 0.97 (95%CI: 0.96–0.98) with significant heterogeneity (I^2^ = 60.5%, *P*_heterogeneity_ < 0.01). As shown in Fig. 2a, we found evidence of a nonlinear relationship between BMI and overall LC risk (*P*_nonlinearity_ < 0.01, I^2^ = 5%). Moreover, this dose-response trend showed a statistically significant decreased risk of developing LC with increasing BMI. Note that several representative point values are presented in Table 3. In a sensitivity analysis iteratively omitting each study from the overall analysis, a significant nonlinear dose-response relationship between BMI and overall risk of LC still remained and the point estimation of BMI did not substantially change, suggesting high level of stability for the current result.

After stratifying by sex, the non-linear relationship between BMI and LC risk was observed in both subgroups (all *P*_nonlinearity_ < 0.05; Fig. 2b,c, respectively). After stratifying by smoking status, it is worthwhile to note that no evidence of a nonlinear association was observed in current smokers, ex-smokers and non-smokers (all *P*_nonlinearity_ > 0.05, Fig. 2d–f, respectively). We also performed a stratified analysis by sex based on smoking status, apart from female current smokers, and a statistically linear association between BMI and LC risk was observed in the rest of the groups (all *P*_nonlinearity_  > 0.05). After stratifying by histologic type, there was a significant nonlinear association between BMI and lung adenocarcinoma risk (*P*_nonlinearity_ < 0.01, Fig. 2g).

For non-smokers, it is noteworthy that the SRRs of LC risk were 0.96 (95%CI: 0.91–1.01) at the point of 30 kg/m^2^, 0.94 (95%CI: 0.81–1.08) at 35 kg/m^2^, and 0.92 (95%CI: 0.72–1.17) at 40 kg/m^2^. For male non-smokers, the SRRs of LC risk were 0.96 (95%CI: 0.86–1.07) at the point of 30 kg/m^2^ and 0.95(95%CI: 0.71–1.27) at 35 kg/m^2^. For female non-smokers, the SRRs of LC risk were 0.95 (95%CI: 0.89–1.02) at the point of 30 kg/m^2^ and 0.95 (95%CI: 0.76–1.10) at 35 kg/m^2^. A sensitivity analysis for non-smokers was also conducted. Excluding those that were not controlled for potential confounders, none of the point estimates were materially altered, indicating that our results were robust.

### Sources of heterogeneity and publication bias

**Figure 3:**
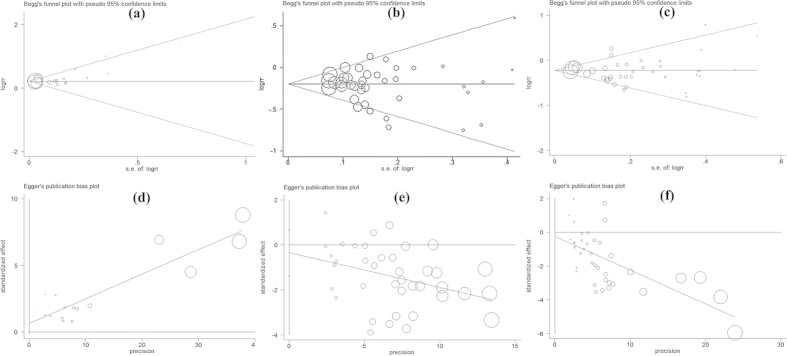
Egger’s publication bias plots and Begg’s funnel plots of the cohort studies that investigated abnormal BMI (underweight, overweight and obesity) and LC risk. Begg’s funnel plot: (**a**) Underweight vs. normal weight; (**b**) Overweight vs. normal weight; (**c**) Obesity vs. normal weight. Egger’s test: (**d**) Underweight vs. normal weight; (**e**) Overweight vs. normal weight; (**f**) Obesity vs. normal weight. Underweight, BMI ≤ 18.5 kg/m^2^; Normal weight, BMI = 18.5–24.9 kg/m^2^; Overweight, BMI = 25–29.9 kg/m^2^; Obesity, BMI ≥ 30 kg/m^2^; BMI, body mass index; LC, lung cancer; S.E., standard error; RR, relative risk; Area of a circle represents the weight of individual study. The pseudo 95%CI is computed as part of the analysis that produces the funnel plot.

For studies of those that were overweight, we found no significant heterogeneity between studies when stratified by the number of cases or study quality scores (Table 2). In multivariate meta-regression analyses, the combination of four variables (including sex, body size assessment, the number of cases and study quality score) was statistically significant. The between-study variance was reduced from 0.008 to 0.001 based on the REML estimate, and the heterogeneity explained by these four variables was 88.24%. In studies of obesity, the number of cases was identified as being a major source of heterogeneity between studies (*P* = 0.04), and the between-study variance was reduced from 0.008 to 0.000. The heterogeneity explained by the number of cases was 98.70%. In addition, the visual inspection of the Begg’s funnel plot or Egger’s plot revealed symmetry (Fig. 3), and there was no significant publication bias detected by the Begg’s and Egger’s tests (*P* = 0.09 for underweight, 0.70 for overweight, 0.23 for obesity).

## Discussion

The present meta-analysis is the first to provide comprehensive and quantitative evidence of the dose-response association between BMI and LC risk. We found that lower BMI was associated with higher LC risk, and each 5 kg/m^2^ increment of BMI corresponded to a 3.3% decrease in the risk of LC. BMI was inversely nonlinearly associated with overall LC risk. The reduction affects men and women alike, and it was greater for obesity than for overweight, displaying a significant nonlinear dose-response relationship. Note that the subgroup results of the dose-response analysis should be interpreted with caution because only a few studies focused on the histologic subtypes of LC, and interestingly, significant linear relationships that might result from heterogeneity and residual confounding were observed in current smokers, ever-smokers and non-smokers, respectively.

One of the previous meta-analyses showed a negative association of 0.79 (95%CI: 0.73–0.85) between the risk of LC and excess weight (BMI ≥ 25 kg/m^2^)^12^. Moreover, Renehan *et al.* reported that a 5 kg/m^2^ increase in BMI was inversely associated with LC risk, and the pooled RRs were 0.76 (95%CI: 0.70–0.83) for men and 0.80 (95%CI: 0.66–0.97) for women^13^. This is in line with our results. As overweight/obesity was closely related to a lower risk of LC, we proposed the hypothesis that being underweight was related to a higher risk of LC. In our meta-analysis, indeed, being underweight was associated with an increased risk of LC. We also found that being underweight might not exert an inverse effect on modulating LC risk in ex-smokers and those that have never smoked. In addition, the results showed no evidence of publication bias in the overall and stratified analyses for underweight, which indicated a good representation of the extracted studies. The observed underweight–LC association in the present study, if validated in future studies, has relevance for public health.

Previously published systematic reviews, investigating overweight/obesity and risk of LC, were both based on the cohort and case-control studies. The inherent limitation was that the cut points for BMI differed between the included studies, and information bias in case-control studies was of particular concern, especially for smoking. It is essential to clarify the shape of the dose-response curve, as inconsistencies in the results between studies relate to different BMI levels. It is possible that only current smokers or ever smokers may benefit from excess weight, and the implications for those individuals with moderate or high BMI status that smoke may be different. Moreover, some uncertainties may be present in the extrapolation from low to high BMI in a nonlinear dose-response association, and therefore, demanding caution for LC prevention. Hence, we conducted a dose-response meta-analysis of cohort studies to evaluate the optimal range of BMI. We found BMI had a negative association with incidence of LC in men and in women. In light of our findings, the current guidelines for lowering the risk of LC among the general population may be revised to recommend 18.5 < BMI < 30 kg/m^2^, and meanwhile, avoiding obesity is also necessary considering that obesity increases the risk of certain cancers and cardiovascular disorders^33^, a relationship that has already been confirmed.

Confounding variables represent a primary issue in observational aetiological studies. The finding that obesity has been associated with a reduced LC risk may be due to the confounding caused by smoking, because smoking habits affect both body weight and body composition^34^,^35^. The general BMI–smoking interaction is considerably complex and is subject to change over time. Importantly, obesity in relation to lung cancer in non-smokers is difficult to interpret. Present study investigating BMI–LC associations has conventionally stratified for smoking status. Strong inverse association was observed between BMI and the risk of lung cancer among former and current smokers. However, the findings from our current dose-response meta-analysis suggest that increased BMI has a negligible effect on the risk of LC in non-smokers, and obesity was not significantly associated with risk of LC. Meanwhile, it appears that the results of the subgroup analysis according to females and males were due to smoking status, particularly among current smokers. Since increased BMI is associated with decreased LC risk in smokers and no association was found in non-smokers, one could, of course, speculate that the decreased risk of LC has been driven less by excess body weigh in non-smokers, or that only smokers benefit more from obesity in terms of lowered risk of LC. It is implied that an effect of BMI on lung cancer is independent of smoking status. Although we cannot rule out the effect of uncontrolled confounding by smoking, We can be certain that the overall impact of BMI–smoking on LC risk is comparably apparent, and suitable body weight as well as the smoking control is by far the very efficient measure to prevent LC.

The theoretical biological plausibility for the negative association between obesity and LC risk is as follows. (1) Possible plausibility exists through the effect of adipose tissue on storage, mobilisation, and metabolism of carcinogen-DNA adducts^36^,^37^. Both 8-hydroxydeoxyguanosine (8-OHdG) and benzo(a)pyrene (BaP) adduct, for example, are the most frequently encountered carcinogen-DNA adducts^38^. Studies had found a significant inverse association between BMI and BaP adduct, urinary levels of 8-OHdG^39^,^40^. (2) Excess body fat is linked to elevated production of insulin, resulting in the increase of insulin–like growth factor I and secretion of sex steroids, which subsequently promotes cell proliferation and suppresses apoptosis, and thus improve immune function and inhibit carcinogenesis^41^,^42^. Moreover, studies that have shown reduced risks of lung cancer associated with the use of menopausal hormones have contributed to speculations that oestrogens may exert beneficial effects against LC^43^,^44^,^45^. Actually, several studies had found a positive association between BMI and serum oestrogen levels, and adipose tissue is the primary site for oestrogen synthesis^45^,^46^. (3) Brennan *et al.* reported that obesity associated with the gene (FTO) A allele, which was proven to correlate with increased BMI, was also associated with a decreased risk of LC^47^,^48^. The biological mechanisms behind such a negative obesity–LC link remains unclear, and thus, better designed molecular–epidemiologic studies and further work in exploring the underlying carcinogenic mechanism are necessary.

A notable strength of the present methodology is the trend estimation of the summarised dose-response data, not only offering uniform analysis of cohort studies with different BMI categories and analysis of studies across varied levels of BMI, but also offering greater power using the full spectrum of continuous BMI data. Several limitations in our review should be mentioned. (1) There are several methodological limitations (including BMI assessment and analytic methodology) that are worth consideration. (2) Unmeasured and residual confounding variables might bias the results toward the exaggeration or underestimation of risk estimates. Because of the inability to fully adjust for confounders, the protective effect of increasing BMI on LC could be attributed to other favourable lifestyle factors. (3) Due to the limitation of eligible data, the subgroups based on smoking status were crudely classified into current smoking, ex-smoking and non-smoking subgroups, regardless of the smoking duration, cumulative smoking exposure, and smoking intensity. (4) High heterogeneity across studies was presented for the overweight–LC and obesity–LC links, which would throw some doubts on the reliability of the SRR estimates. (5) Only studies published in the electronic edition of the databases in the English and Chinese languages have been reviewed for pertinence and studies in other languages were omitted. Despite these limitations, the meta-analysis included a substantial number of cohort studies, which greatly enhanced the statistical power of the analysis and provided enough evidence for the authors to draw a reliable conclusion.

In summary, our meta-analysis contributes to the literature by exploring the nonlinear dose-response relationship between BMI and the risk of LC. An inverse dose-response relationship is likely to be present for LC risk with increasing BMI. Being underweight was strongly associated with increased risk of LC. Excess weight provides a potential protective effect against LC. However, the results from dose-response analyses in non-smokers support the idea that BMI has a modifying effect on smoking-related LC risk. Studies with large-scale, longer follow-up times and studies that are well-designed are warranted to confirm these subgroup results.

## Additional Information

**How to cite this article**: Duan, P. *et al.* Body mass index and risk of lung cancer: Systematic review and dose-response meta-analysis. *Sci. Rep.* **5**, 16938; doi: 10.1038/srep16938 (2015).

## Supplementary Material

Supplementary Information

## Figures and Tables

**Table 1:** Stratified meta-analyses of underweight (BMI < 18.5 kg/m^2^) and the risk of lung cancer.

Studies groups	N	RR (95% CI)	Heterogeneity		M	E-T
			*I*^2^	*P*		
*All studies*	14	1.24(1.20–1.27)	24.3	0.17	F	0.09
*Sex*						
Male	6	1.25(1.21–1.29)	31.7	0.19	F	0.33
Female	6	1.20(1.13–1.27)	47.0	0.09	R	0.09
*Geographic location*						
Europe	3	1.24(1.20–1.27)	9.6	0.48	F	0.23
North America	4	1.21(1.15–1.27)	32.1	0.23	F	0.20
Asia	7	1.28(1.23–1.34)	0.0	0.45	F	0.08
*Body size assessment*						
Measured	11	1.25(1.21–1.29)	1.2	0.44	F	0.28
Self-reported	3	1.19(1.11–1.27)	67.6	0.05	R	0.24
*Smoking status*						
Current smokers	4	1.31(1.10–1.57)	0.0	0.78	F	0.68
Ex-smokers	4	1.40(0.82–2.36)	70.5	0.01	R	0.21
Non-smokers	5	1.18(0.90–1.54)	0.0	0.91	F	0.19
*Number of cases*						
<500	4	1.27(1.21–1.34)	46.6	0.11	F	0.15
500–1000	4	1.21(1.15–1.27)	26.1	0.26	F	0.25
≥1000	6	1.23(1.175–1.29)	10.4	0.35	F	0.94
*Duration of follow-up*						
<10 years	7	1.19(1.12–1.26)	0.0	0.67	F	0.16
≥10 years	7	1.24(1.20–1.27)	42.6	0.08	R	0.09
*Adjustment for age, smoking and education*						
Yes	4	1.30(1.12–1.51)	22.3	0.27	F	0.12
No	10	1.23 (1.20–1.27)	29.5	0.16	F	0.25
*Adjustment for age, smoking and physical activity*						
Yes	7	1.25(1.21–1.30)	0.0	0.70	F	0.29
No	7	1.21(1.15–1.27)	46.6	0.06	R	0.13
*Adjustment for age, smoking and alcohol consumption*						
Yes	8	1.25(1.21–1.29)	31.2	0.14	F	0.41
No	6	1.21(1.15–1.27)	24.3	0.41	F	0.11

Note: All the groups were compared with normal weight (18.5 ≤ BMI ≤ 24.9 kg/m^2^) as the reference category. N: Number of the studies; M: Statistical method; R: Random model; F: Fixed model; E–T: Egger’s test

**Table 2:** Subgroup analyses of excess weight (BMI≥25 kg/m^2^) and the risk of lung cancer.

Studies groups	Overweight (25≤BMI≤29.9 kg/m^2^)					Obesity (BMI≥30 kg/m^2^)				
	N	RR (95% CI)	Heterogeneity		E-T	N	RR (95% CI)	Heterogeneity		E–T
			I^2^	*P*				I^2^	*P*	
*All studies*	29	0.82(0.77–0.86)	35.7	0.02	0.51	29	0.78(0.74–0.83)	40.3	0.01	0.457
*Sex*										
Male	16	0.78(0.72–0.85)	41.3	0.04	0.27	16	0.77(0.70–0.84)	40.1	0.05	0.20
Female	13	0.88(0.79–0.9835)	35.3	0.10	0.55	13	0.78(0.71–0.86)	32.2	0.125	0.35
*Geographic location*										
Europe	10	0.82(0.76–0.89)	0.0	0.92	0.79	10	0.80(0.75–0.86)	0.0	0.71	0.48
North America	11	0.84(0.78–0.91)	22.1	0.21	0.52	11	0.79(0.74–0.84)	16.9	0.27	0.40
Asia	8	0.79 (0.67–0.93)	71.2	0.00	0.54	8	0.74(0.59–0.93)	65	0.00	0.39
*Body size assessment*										
Measured	21	0.84(0.79–0.89)	32.8	0.05	0.56	21	0.79(0.74–0.84)	19.9	0.18	0.39
Self-reported	8	0.73(0.62–0.86)	42.5	0.12	0.40	8	0.71(0.58–0.88)	64.7	0.01	0.29
*Smoking status*										
Current smokers	9	0.79(0.71–0.87)	49.9	0.03	0.06	9	0.72(0.66–0.78)	6.4	0.38	0.05
Ex-smokers	10	0.91(0.85–0.98)	51.0	0.02	0.07	10	0.77(0.69–0.85)	22.9	0.22	0.07
Non-smokers	14	0.86 (0.78–0.94)	40.6	0.04	0.99	12	0.86(0.75–0.98)	22.8	0.21	0.13
*Histologic type*										
Adenocarcinoma	4	0.92 (0.85–0.99)	35.8	0.17	0.31	4	0.79(0.71–0.88)	0.0	0.74	0.25
Small cell carcinoma	3	1.04(0.91–1.18)	29.9	0.23	0.96	3	0.84(0.56–1.26)	63.0	0.03	0.57
Squamous cell carcinoma	4	0.88(0.79–0.98)	34.9	0.19	0.07	4	0.76(0.63–0.92)	0.0	0.83	0.05
*Number of cases*										
<200	5	0.75(0.58–0.96)	36.7	0.15	0.08	5	0.79(0.58–1.08)	22.6	0.25	0.69
200–500	7	0.71(0.61–0.81)	33.8	0.17	0.52	7	0.66(0.58–0.75)	0.0	0.72	0.32
500–1000	8	0.89(0.81–0.97)	24.3	0.23	0.07	8	0.67(0.58–0.77)	0.0	0.83	0.80
≥1000	9	0.85(0.80–0.90)	0.0	0.48	0.47	9	0.83(0.78–0.88)	36.1	0.10	0.62
*Duration of follow-up*										
<10 years	12	0.76(0.68–0.85)	52.7	0.02	0.40	12	0.68(0.60–0.78)	28.3	0.17	0.75
≥10 years	17	0.85(0.80–0.91)	18.8	0.20	0.50	17	0.81(0.76–0.85)	20.4	0.19	0.49
*Study quality score*										
6 sars	3	1.10(0.88–1.39)	0.0	0.45	0.95	3	0.65(0.46–0.91)	0.0	0.79	0.92
7 stars	8	0.67(0.59–0.79)	39.2	0.11	0.66	8	0.66(0.55–0.78)	23.3	0.23	0.07
8 stars	12	0.82(0.77–0.88)	0.0	0.62	0.81	12	0.86(0.80–0.92)	0.0	0.47	0.82
9 stars	6	0.88(0.82–0.94)	0.0	0.73	0.31	6	0.77(0.73–0.82)	31.9	0.17	0.81
*Adjustment for age, smoking and education*										
Yes	9	0.82(0.76–0.88)	2.7	0.42	0.51	9	0.80(0.75–0.85)	14.7	0.31	0.10
No	20	0.82(0.78–0.87)	45.8	0.01	0.86	20	0.76(0.69–0.84)	39.9	0.02	0.70
*Adjustment for age, smoking and physical activity*										
Yes	10	0.82(0.74–0.91)	58.3	0.00	0.83	10	0.79(75–0.84)	28.6	0.16	0.13
No	19	0.82(0.77–0.88)	15.4	0.26	0.89	19	0.77(0.69–0.85)	34.7	0.05	0.69
*Adjustment for age, smoking and alcohol consumption*										
Yes	12	0.80(0.71–0.89)	54.4	0.01	0.74	12	0.80(0.74–0.86)	29.5	0.15	0.05
No	17	0.84(0.78–0.89)	23.5	0.15	0.76	17	0.76(0.70–0.83)	31.2	0.08	0.97

Note: All the groups were compared with normal weight (18.5 ≤ BMI ≤ 24.9 kg/m^2^) as the reference category. N: Number of the studies; E–T: Egger’s test.

**Table 3:** Risk estimates for lung cancer associated with BMI in stratified analysis.

Studies groups	No. of studies	BMI (kg/m^2^)†					*P*‡	I^2^ (95%CI)
		22.5	25.0	27.5	30	35		
*All studies*	26	1.00(0.99–1.00)	0.98(0.95–1.01)	0.95(0.90–1.00)	0.91(0.85–0.98)	0.81(0.72–0.91)	0.000	5(0–28)
*Sex*								
Male	15	1.00(1.00–1.00)	0.98(0.98–0.99)	0.96(0.95–0.96)	0.92(0.91–0.93)	0.83(0.80–0.86)	0.000	14(0–42)
Female	9	1.00(1.00–1.00)	0.98(0.98–0.99)	0.97(0.96–0.97)	0.94(0.93–0.96)	0.88(0.84–0.93)	0.042	28(0–56)
*Smoking status*								
*Current smokers*	11	0.99(0.99–0.99)	0.97(0.97–0.97)	0.95(0.94–0.95)	0.92(0.91–0.94)	0.87(0.83–0.91)	0.468	35(0–65)
Male	5	0.99(0.99–0.99)	0.97(0.97–0.98)	0.95(0.95–0.96)	0.93(0.92–0.95)	0.88(0.82–0.96)	0.921	0(0–60)
Female	6	1.00(1.00–1.00)	0.98(0.98–0.99)	0.96(0.95–0.97)	0.92(0.89–0.94)	0.81(0.75–0.87)	0.000	0(0–46)
*Ex-smokers*	8	1.00(0.99–1.00)	0.98(0.98–0.99)	0.97(0.96–0.98)	0.95(0.93–0.97)	0.89(0.84–0.94)	0.076	38(6–59)
Male	5	1.00(1.00–1.00)	0.99(0.98–1.00)	0.97(0.96–0.99)	0.95(0.92–0.98)	0.89(0.82–0.97)	0.198	40(0–68)
Female	5	1.00(0.99–1.00)	0.98(0.97–1.00)	0.97(0.95–0.98)	0.94(0.91–0.97)	0.88(0.81–0.96)	0.230	43(1–67)
*Non-smokers*	12	1.00(1.00–1.00)	0.98(0.97–0.99)	0.97(0.94–0.99)	0.96(0.91–1.01)	0.94(0.81–1.08)	0.815	27(0–50)
Male	7	1.00(1.00–1.00)	0.98(0.97–1.00)	0.97(0.93–1.01)	0.96(0.86–1.07)	0.95(0.71–1.27)	0.830	37(0–66)
Female	6	1.00(1.00–1.00)	0.98(0.96–1.01)	0.97(0.94–1.00)	0.95(0.89–1.02)	0.92(0.76–1.10)	0.850	23(0–56)
*Histologic type*								
Adenocarcinoma	5	1.00(1.00–1.00)	0.99(0.98–0.99)	0.96(0.95–0.98)	0.92(0.90–0.95)	0.82(0.76–89)	0.001	10(0–46)
Small cell carcinoma	3	1.00(1.00–1.00)	0.99(0.98–1.01)	0.99(0.97–1.01)	0.99(0.97–1.02)	1.00(0.92–1.08)	0.774	0(0–57)
Squamous cell carcinoma	4	1.00(0.99–1.00)	0.98(0.97–0.99)	0.97(0.95–0.99)	0.96(0.90–0.99)	0.90(0.80–1.01)	0.629	27(0–60)

Note:

^†^The association between BMI and lung cancer risk was evaluated by relative risk (RR) and 95% confidence interval (CI). Besides, normal weight (BMI = 18.5–24.9 kg/m^2^) was regarded as a reference category.

^‡^*P* value for non-linearity.
