# Supplementary material for: Body mass index and risk of lung cancer: Systematic review and dose-response meta-analysis
Source: Sci Rep. 2015 Nov 19;5:16938. doi: 10.1038/srep16938 (PMC4652238; doi:10.1038/srep16938)
Supplement: Supplementary Information [file srep16938-s1.pdf]

The title of the paper

Body mass index and risk of lung cancer: Systematic review and dose-response meta-analysis

The full names of the authors

Peng Duan[1], Chunhui Hu[2], Chao Quan[1], Xianfu Yi[3], Wei Zhou[1], Meng Yuan[1], Tingting Yu[1], Ansoumane Kourouma[1] & Kedi Yang[1*]

Affiliations of authors

[1]MOE (Ministry of Education) key lab of Environment and Health, Department of Occupational and Environmental Health, Tongji Medical College, Huazhong University of Science and Technology, Wuhan, Hubei, China;

[2]Department of Clinical Laboratories, the Affiliated Taihe Hospital, Hubei University of Medicine, Shiyan, Hubei, China;

[3]Department of Emergency, the Affiliated Taihe Hospital, Hubei University of Medicine, Shiyan, Hubei, China;

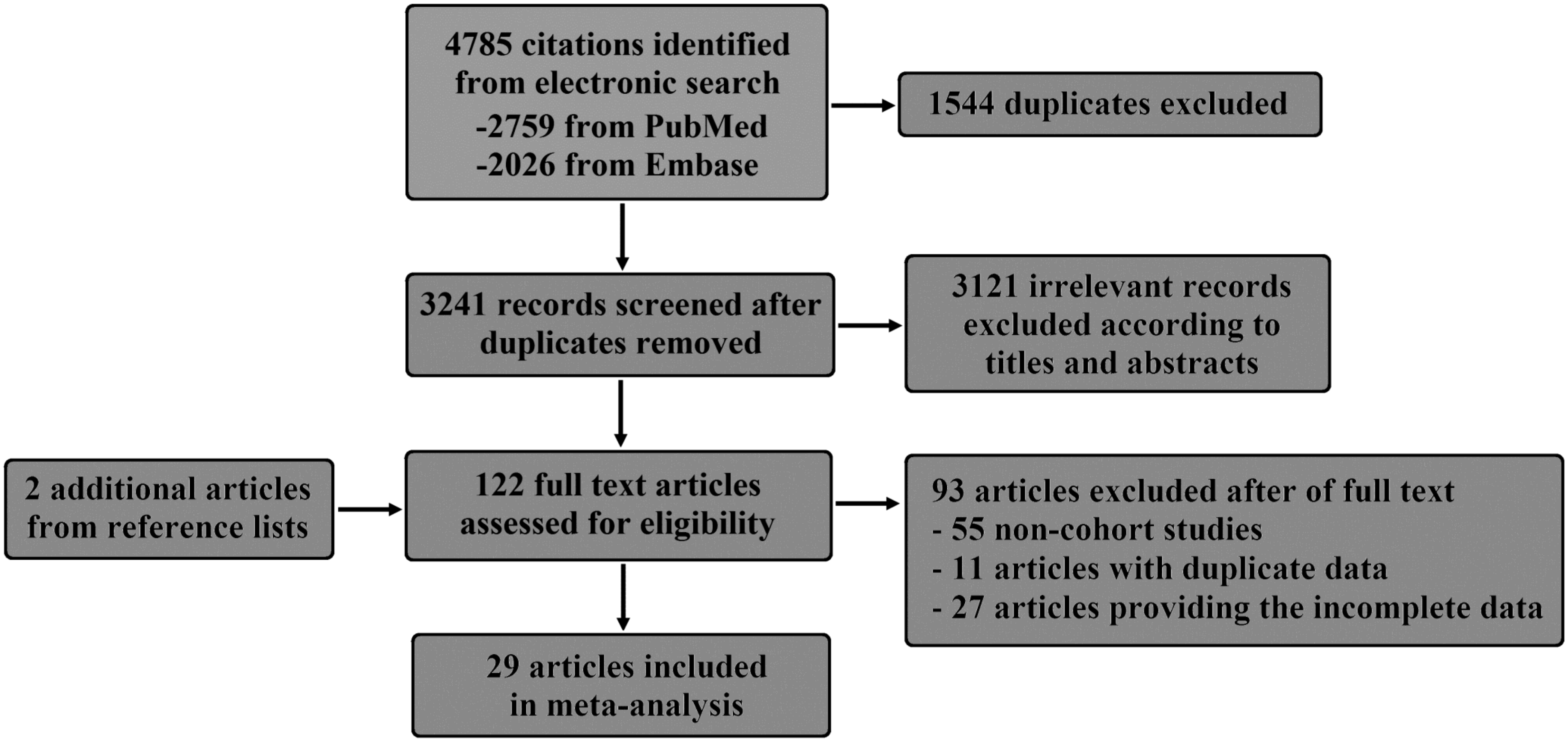

Figure S1 Flow diagram of literature search and study selection.

Table S1. Characteristics of cohort studies included in meta-analysis

First author, year, country reference	Study participants, n	Study period (including follow-up)	Sex and age (mean or range)	Case ascertainment	Number of cases	Anthropometric measurement	BMI categories (kg/m^2)	Relative risk with 95% CI	Main Confounders adjusted†
Guo L, 2014, China[1]	Chinese cohort of Kailuan Company, n=133,273	2006-2011	Men, 51.07±13.54 yrs	Cancer registry	386	Direct measured	<18.5<br>18.5-24.0<br>24.0-28.0<br>>28.0	1.38(0.82-2.35);<br>1;<br>0.62(0.46-0.76);<br>0.69(0.44-0.92)	1, 2, 3, 5
Everatt R, 2014, Lithuania[2]	Kaunas-Rotterdam Intervention Study and Multifactorial ischemic heart disease prevention study, n=6,729	1978–2008	Men, 52.7+5.7 yrs	Cancer registry	358	Direct measured	<25.0<br>25.0-29.9<br>>30.0	1;<br>0.81(0.65-1.02);<br>0.69(0.49-0.97)	1, 2, 3, 5
Bethea TN, 2013, USA [3]	The Black Women's Health Study, n=56,835	1995-2011	Women, mean age in 1995 was 38.6 yrs	Cancer registry	323	Self-reported	<18.5<br>18.5-24.9<br>25.0-29.0<br>≥30	2.7(1.36-5.42);<br>1;<br>0.85(0.65-1.11);<br>0.69(0.52-0.93)	1, 2, 3, 4, 5, 9, 11, 13, 15
Hatlen P, 2013, Norway [4]	The Nord-Trøndelag Health Study, n= 6,996	1996-2008	Men and women, 68 y ±9 yrs	Cancer registry	132	Direct measured	Women<br><18.5<br>18.5-24.9<br>25.0-29.9<br>≥30<br>Men<br><18.5<br>18.5-24.9<br>25.0-29.9<br>≥30	Women<br>1.02(0.14-7.59);<br>1;<br>0.49(0.29-0.81);<br>0.54(0.31-0.95)<br>Men<br>5.16(1.39-19.10);<br>1;<br>0.83(0.48-1.41);<br>0.67(0.32-1.38)	1, 2
Lam TK, 2013, USA [5]	National Institute of Health (NIH)-AARP Diet and Health Study, n=337,074	1995-2006	Men and women, 50-71 yrs	Cancer registry	532	Direct measured	<18.5<br>18.5-25.0<br>25.0-30.0<br>≥30	1.57(0.77-3.19);<br>1;<br>1(0.81-1.22);<br>1.21(0.95-1.53)	1, 3, 4, 5, 7, 17

Smith L, 2012, USA [6]	National Institutes of Health–AARP Diet and Health Study, n=448,732	1995-2006	Men and women, 50-71 yrs	Cancer registry	9415	Direct measured	Men<br><18.5<br>18.5-22.49<br>22.5-24.99<br>25-29.99<br>30-34.99<br>>35<br>Women<br><18.5<br>18.5-22.49<br>22.5-24.99<br>25-29.99<br>30-34.99<br>>35	Men<br>1.15(0.83-1.59);<br>1.12(1.02-1.23);<br>1;<br>0.92(0.86-0.99);<br>0.87(0.80-0.98);<br>0.81(0.7-1.0)<br>Women<br>1.23(0.97-1.54);<br>1.15(1.04-1.26);<br>1;<br>0.99(0.9-1.08);<br>0.85(0.75-0.96);<br>0.73(0.61-0.87)	1, 2, 3, 4, 5, 7, 12
Leung CC, 2010, China [7]	The Elderly Health Service, n=64,574	2000-2008	Men and women, ≥65 yrs	Cancer registry	932	Direct measured	<18.5<br>18.5-23.0<br>23.0-25.0<br>25.0-30.0<br>>30	1.20(0.86-1.68);<br>1;<br>0.82(0.66-1.01);<br>0.83(0.68-1.00);<br>0.49(0.31-0.79)	1, 2, 5, 6, 26
Dehal A, 2011, USA [8]	National Health and Nutrition Examination Survey, n=14,407	1971-1992	Women, 25-47 yrs	Cancer registry	124	Direct measured	18.5-24.9<br>25.0-29.9<br>≥30	1;<br>0.87(0.59-1.28);<br>0.98(0.57-1.70)	2, 5, 6, 7, 15, 16, 20, 26
Parr CL, 2010, Norway [9]	The Asia-Pacific Cohort Studies Collaboration, n=424,519	1961-1999	Men and women, mean age was 48 yrs	Cancer registry	1478	Direct measured	<18.5<br>18.5-25.0<br>25.0-30.0<br>≥30	1.11(0.86-1.44);<br>1(0.92-1.09);<br>0.68(0.59-0.79);<br>0.83(0.64-1.08)	1, 2, 5

Andreotti G, 2010, USA [10]	Details of the Agricultural Health Study, n=57,310	1993-2005	Men and women, 40-69 yrs	Cancer registry	357	Self-reported	Men<br>18.5-25.0<br>25.0-30.0<br>30-35<br>>35<br>Women<br>18.5-25.0<br>25.0-30.0<br>30.0-35.0	Men<br>1;<br>0.85(0.60-1.2);<br>0.85(0.54-1.35);<br>0.47(0.15-1.49)<br>Women<br>1;<br>0.99(0.63-1.55);<br>0.67(0.33-1.38)	2, 4, 7, 15, 20
Koh WP, 2010, China [11]	The Singapore Chinese Health Study, n=63,257	1993-2006	Men and women, 45-74 yrs	Cancer registry	1042	Self-reported	<20<br>20-24<br>24-28<br>≥28	1.34(0.98-1.83);<br>1;<br>0.91(0.66-1.25);<br>0.90(0.65-1.25)	1, 2, 3, 6, 9, 10
Song YM, 2008, Korea [12]	Postmenopausal Korean women, n=170,481	1994–2003	Women, 40-64 yrs	Cancer registry	621	Direct measured	<18.5<br>18.5-20.9<br>21.0-22.9<br>23.0-24.9<br>25.0-29.9<br>≥30	1.80(1.18-2.73);<br>1.34(1.00-1.18);<br>1;<br>1.05(0.81-1.35);<br>1.17(0.90-1.52);<br>0.48(0.24-0.94)	1, 2, 4, 5, 6, 16
Kabat GC, 2008, USA [13]	The Women’s Health Initiative, n=161,809	1980–2000	Women, 50-79 yrs	Pathology reports	1365	Direct measured	<23.1<br>23.1-25.6<br>25.6-28.3<br>28.3-32.2<br>≥32.2	1;<br>1.04(0.88-1.23);<br>0.77 (0.64-0.93);<br>0.81(0.68-0.98);<br>0.79(0.65,0.96)	1, 2, 3, 4, 5, 6, 7, 9, 17, 19, 20, 23
Jee SH, 2008, Korea [14]	Korean Cancer Prevention Study, n=1,329,525	1992-2006	Men and women, 30-95 yrs	Cancer registry	11297	Direct measured	Men<br><20.0<br>20.0-22.9<br>23.0-24.9<br>25.0-29.9<br>≥30<br>Women<br><20.0<br>20.0-22.9<br>23.0-24.9<br>25.0-29.9<br>≥30	Men<br>1.35(1.24-1.47);<br>1.13(1.05-1.21);<br>1;<br>0.92(0.84-1.0);<br>1.29(0.96-1.73)<br>Women<br>1.20(1.00-1.44);<br>1.18(1.02-1.37);<br>1;<br>1.1(0.94-1.29);<br>0.91(0.63-1.33)	1, 2, 4, 5
Kabat GC, 2007, USA [15]	Canadian National Breast Screening Study, n=19,352	1980-2000	Women, 40-59 yrs	Cancer registry	520	Direct measured	Never smokers<br><21.6<br>21.6-23.3	Never smokers<br>1;<br>0.91(0.36-2.31);<br>1.97(0.88-4.41);	1, 2, 3, 6, 9, 10

							23.3-25.1<br>25.1-27.9<br>≥27.9<br>Current smokers<br><21.6<br>21.6-23.3<br>23.3-25.1<br>25.1-27.9<br>≥27.9<br>Ex-smokers<br><21.6<br>21.6-23.3<br>23.3-25.1<br>25.1-27.9<br>≥27.9	1.81(0.80-4.06);<br>2.19(1.0-4.8)<br>Current smokers<br>1;<br>0.77(0.60-0.99);<br>0.77(0.60-1.00);<br>0.64(0.49-0.85);<br>0.63(0.48-0.83)<br>Ex-smokers<br>1;<br>1.06(0.63-1.80);<br>0.65(0.36-1.16);<br>0.47(0.25-0.88);<br>0.69(0.39-1.23)	
Reeves GK, 2007, UK [16]	The million women study, n=1,222,630	1996-2001	Women, 50-64 yrs	Cancer registry	3171	Self-reported	<22.5<br>22.5-24.9<br>25.0-27.4<br>27.5-29.5<br>≥30	1.17(1.09-1.25);<br>1;<br>0.91(0.85-0.99);<br>0.83(0.75-0.91);<br>0.84(0.77-0.92)	1, 2, 4, 5, 6, 11, 15, 16, 23
Samanic C, 2006, Sweden [17]	Workers in the construction industry, n=362,552	1971-1999	Men, 18-67 yrs	Cancer registry	2831	Direct measured	18.5-24.9<br>25.0-29.9<br>≥30	1;<br>0.80(0.74-0.87)<br>0.74(0.63-0.88)	1, 2, 6
Lukanova A, 2006, Sweden [18]	The Northern Sweden Health and Disease Cohort, n=74,207	1985-2003	Men and women, 30-60 yrs	Cancer registry	95	Direct measured	Men<br>18.5-23.4<br>23.5-25.3<br>25.4-27.6<br>≥27.7<br>Women<br>18.5-22.1<br>22.2-24.2<br>24.3-27.0<br>≥27.1	Men<br>1;<br>0.84(0.41-1.73);<br>0.84(0.44-1.78)<br>0.72(0.34-1.49)<br>Women<br>1;<br>0.85(0.36-1.98);<br>0.97(0.44-2.20);<br>1.26(0.6-2.75)	1, 2
Batty GD, 2005, UK [19]	The Whitehall Study, n=18,403	1967-2002	Men, 40-64 yrs	Cancer registry	783	Direct measured	18.5-24.9<br>25.0-29.9<br>≥30	1;<br>0.85(0.73-0.98)<br>0.77(0.51-1.15)	1, 4, 5, 6

Kuriyama S, 2005, Japan [20]	Residents in 3 municipalities of Miyagi Prefecture, n=27,539	1984-1992	Men and women,≥40 yrs	Cancer registry	193	Self-reported	Women<br>18.5-24.9<br>25.0-27.4<br>27.5-29.9<br>≥30<br>Men<br>18.5-24.9<br>25.0-27.4<br>27.5-29.9<br>≥30	Women<br>1;<br>0.39(0.14-1.10);<br>0.44(0.10-1.82);<br>1.72(0.6-4.91)<br>Men<br>1;<br>0.77(0.47-1.26);<br>0.30(0.07-1.20);<br>0.29(0.20-3.26)	1, 2, 5, 11, 18, 19, 20, 26
Rapp K, 2005, Austria [21]	The Vorarlberg Health Monitoring and Promotion Program, n=145,931	1985-2002	Men and women, 35-54 yrs	Cancer registry	590	Direct measured	Men<br>18.5-24.9<br>25.0-29.9<br>30.0-34.9<br>≥35<br>Women<br>18.5-24.9<br>25.0-29.9<br>30.0-34.9	Men<br>1;<br>0.80(0.66-0.97);<br>0.80(0.65-1.20)<br>0.79(0.41-1.86)<br>Women<br>1;<br>0.99 (0.68-1.48);<br>0.87(0.50-1.5)	1, 2, 21
Oh SW, 2005, Korea [22]	Korea National Health Insurance Corporation, n=781,283	1992-2001	Men, ≥20 yrs	Cancer registry	2264	Direct measured	<18.5<br>18.5-22.9<br>23.0-24.9<br>25.0-26.9<br>27.0-29.9<br>≥30	1.26(0.98-1.61);<br>1;<br>0.88(0.80-0.97);<br>0.80(0.70-0.90)<br>0.78(0.65-0.94)<br>0.77(0.47-1.30)	1, 2, 4, 5, 6, 14, 15
Liu EJ, 2004, China [23]	Male residents of urban Shanghai, n=18,244	1986-2003	Men, 45-64 yrs	Cancer registry	467	Self-reported	<19.5<br>19.5-21.1<br>21.2-22.7<br>22.8-24.5<br>≥24.6	1;<br>0.8(0.6-1.1);<br>0.8(0.6-1.1);<br>0.6(0.4-0.9);<br>0.60(0.50-0.90)	1, 3, 5, 6, 8, 9, 12
Calle EE, 2003, USA [24]	Cancer Prevention Study II, n=1,184,617	1982-1998	Men and women, ≥45 yrs	Cancer registry	15255	Direct measured	Men<br>18.5-24.9<br>25.0-29.9<br>30.0-34.9<br>≥35<br>Women<br>18.5-24.9<br>25.0-29.9<br>30.0-34.9<br>35.0-39.9<br>≥40	Men<br>1;<br>0.78(0.75-0.82);<br>0.78(0.73-0.86);<br>0.67(0.54-0.84)<br>Women<br>1;<br>0.88(0.83-0.94);<br>0.82(0.72-0.92);<br>0.66(0.50-0.86);<br>0.66(0.52-1.28)	1, 2, 3, 4, 5, 7, 19, 20, 23, 26

Olson JE, 2002, USA [25]	Iowa Women’s Health Study, n=38,006	1986-1998	Women, 55-69 yrs	pathology reports	532	Self-reported	≤22.89<br>22.90-25.04<br>25.05-27.43<br>27.44-30.69<br>≥30.70	1;<br>0.92(0.73-1.16);<br>0.76(0.58-0.98);<br>0.69(0.52-0.90);<br>0.66(0.50-0.89)	1, 2, 3, 4, 5, 6, 9
Drinkard CR, 1995, USA [26]	Women with a valid Iowa driver's license, n=41,837	1986-1992	Women, 55-69 yrs	Cancer registry	233	Self-reported	≤23.45<br>23.45-26.11<br>26.12-29.68<br>≥29.68	1;<br>0.54(0.38-0.77);<br>0.52(0.37-0.74));<br>0.52(0.36-0.74)	1, 2, 3, 4, 5, 6, 9, 10
Kark JD, 1995, Israel [27]	The Israel Civil Servant Study, n=9,975	1963-1986	Men, 40-69 yrs	Cancer Registry	153	Direct measured	≥28.32<br>26.44-28.31<br>24.82-26.43<br>22.93-24.81<br>22.27-22.92<br>21.38-22.26<br>20.18-21.37<br><20.18	1;<br>1.10(0.60-2.02);<br>1.09(0.60-1.99);<br>1.27(0.72-2.24);<br>1.36(0.62-2.98);<br>2.26(1.14-4.48);<br>2.78(1.44-5.38);<br>2.85(1.51-5.35)	1, 2, 4, 5, 6, 11, 15, 16, 23
Chyou PH, 1994, USA [28]	Honolulu Heart Program, n=7,840	1965-1988	Men, for age not given	Cancer registry	236	Direct measured	≤22.0<br>22.0-23.9<br>24.0-25.9<br>≥26.0	1;<br>0.85(0.60-1.20);<br>0.85(0.67-1.33);<br>0.69(0.46-1.02)	1, 2, 6, 10
Knekt P, 1991, Finland [29]	The Social Insurance Institution's Mobile Clinic Unit, n=25,994	1966-1984	Men, 20-75 yrs	Cancer registry	504	Direct measured	≥27.0<br>25.1-27.0<br>22.6-25.0<br><22.5	1;<br>1.4(1.0-1.9);<br>1.5(1.2-2.0);<br>1.8(1.4-2.4)	1, 2, 6, 12, 15, 16

†Main confounders adjusted for: 1, age; 2, smoking; 3, education; 4, physical activity; 5, alcohol consumption; 6, gender; 7, ethnicity; 8, age started smoking; 9, pack-years of smoking; 10, number of years since quitting smoking for former smoker; 11, reproductive history; 12, previous chronic bronchitis; 13, family history of lung cancer; 14, family history of cancer; 15, residency area; 16, socioeconomic status; 17, calories intake; 18, fish consumption; 19, fat intake; 20, fruit and vegetable consumption; 21, occupational exposures; 22, exposure to cooking oil fumes; 23, replacement therapy; 24, hypertension; 25, age at menarche; 26, marital status. BMI, body mass index; n, number of study participants; yrs, years; CI, confidence interval.

Table S2. Methodological quality of studies included in the meta-analysis*

**First author, year, country** reference	**Representativeness of the exposed cohort**	**Selection of the unexposed cohort**	**Exposure assessments**	**Outcome of interest not present at the start of the study**	**Control for important factors or additional factors†**	**Outcome assessments**	**Follow-up period long enough for outcomes to occur‡**	**Adequacy of follow-up of cohorts§**	**Total quality scores**
Guo L, 2014, China [1]	★	★	★	★	★★	★	—	—	7
Everatt R, 2014, Lithuania [2]	★	★	★	★	★★	★	★	★	9
Bethea TN, 2013, USA [3]	—	★	★	★	★★	★	★	★	8
Hatlen P, 2013, Norway [4]	—	★	★	★	★★	—	★	—	6
Lam TK, 2013, USA [5]	★	★	★	★	★★	—	★	★	8
Smith L, 2012, USA [6]	★	★	★	★	★★	★	★	★	9
Leung CC, 2010, China [7]	★	★	★	★	★★	★	—	—	7
Dehal A, 2011, USA [8]	★	★	★	★	★★	—	★	★	8
Parr CL, 2010, Norway [9]	—	★	★	★	★★	★	★	★	8
Andreotti G, 2010, USA [10]	—	★	★	★	★★	★	★	—	7
Koh WP, 2010, China [11]	★	★	★	★	★★	★	—	★	8
Song YM, 2008, Korea [12]	—	★	★	★	★★	★	—	—	6
Kabat GC, 2008, USA [13]	★	★	★	★	★★	★	★	★	9
Jee SH, 2008, Korea [14]	★	★	★	★	★★	★	★	★	9
Kabat GC, 2007, USA [15]	—	★	★	★	★★	—	★	—	6
Reeves GK, 2007, UK [16]	★	★	★	★	★★	★	★	★	9
Samanic C, 2006, Sweden [17]	—	★	★	★	★★	★	★	★	8
Lukanova A, 2006, Sweden [18]	★	★	★	★	★★	—	★	—	7
Batty GD, 2005, UK [19]	★	★	★	★	★★	★	★	★	9
Kuriyama S, 2005, Japan [20]	★	★	★	★	★★	—	★	—	7
Rapp K, 2005, Austria [21]	★	★	★	★	★★	★	★	—	8
Oh SW, 2005, Korea [22]	★	★	★	★	★★	★	—	★	8
Liu EJ, 2004, China [23]	—	★	★	★	★★	—	★	★	7
Calle EE, 2003, USA [24]	—	★	★	★	★★	★	★	★	8
Olson JE, 2002, USA [25]	★	★	★	★	★★	★	★	—	8
Drinkard CR, 1995, USA [26]	★	★	★	★	★★	★	—	—	7
Kark JD, 1995, Israel [27]	—	★	★	★	★★	—	★	★	7
Chyou PH, 1994, USA [28]	—	★	★	★	★★	★	★	★	8

Knekt P, 1991, Finland [29]	—	★	★	★	★★	★	★	★	8

[*] A study can be awarded a maximum of one star for each item, except for the item ‘control for important factors or additional factors’.

†A maximum of two stars can be awarded for this item. Studies controlling for BMI : hip ratio were awarded one star, while studies controlling for other important confounders including smoking, age, physical activity and alcohol consumption were awarded an additional star.

‡Duration of 10 years was the predefined follow-up duration long enough for outcomes to occur, and a cohort study with follow-up duration ≥10 years was awarded one star.

§A cohort study with a follow-up rate of more than 75% was awarded one star.

References

1. Guo, L. et al. [Body mass index and cancer incidence:a prospective cohort study in northern China]. *Zhonghua liu xing bing xue za zhi = Zhonghua liuxingbingxue zazhi* **35**, 231-236 (2014).
2. Everatt, R., Virviciute, D., Kuzmickiene, I. & Tamosiunas, A. Body mass index, cholesterol level and risk of lung cancer in Lithuanian men. *Lung cancer* **85**, 361-365 (2014).
3. Bethea, T.N. et al. Obesity in relation to lung cancer incidence in African American women. *Cancer causes & control : CCC* **24**, 1695-1703 (2013).
4. Hatlen, P., Langhammer, A., Forsmo, S., Carlsen, S.M. & Amundsen, T. Bone mass density, fracture history, self-reported osteoporosis as proxy variables for estrogen and the risk of non-small-cell lung cancer--a population based cohort study, the HUNT study: are proxy variables friends or faults? *Lung cancer* **81**, 39-46 (2013).
5. Lam, T.K. et al. Anthropometric measures and physical activity and the risk of lung cancer in never-smokers: a prospective cohort study. *PloS one* **8**, e70672 (2013).
6. Smith, L. et al. Body mass index and risk of lung cancer among never, former, and current smokers. *Journal of the National Cancer Institute* **104**, 778-789 (2012).
7. Leung, C.C. et al. Lower lung cancer mortality in obesity. *International journal of epidemiology* **40**, 174-182 (2011).
8. Dehal, A. et al. Body mass index and death rate of colorectal cancer among a national cohort of U.S. adults. *Nutrition and cancer* **63**, 1218-1225 (2011).
9. Parr, C.L. et al. Body-mass index and cancer mortality in the Asia-Pacific Cohort Studies Collaboration: pooled analyses of 424 519 participants. *Lancet Oncology* **11**, 741-752 (2010).
10. Andreotti, G. et al. Body mass index, agricultural pesticide use, and cancer incidence in the Agricultural Health Study cohort. *Cancer causes & control : CCC* **21**, 1759-1775 (2010).
11. Koh, W.P., Yuan, J.M., Wang, R., Lee, H.P. & Yu, M.C. Body mass index and smoking-related lung cancer risk in the Singapore Chinese Health Study. *British journal of cancer* **102**, 610-614 (2010).
12. Song, Y.M., Sung, J. & Ha, M. Obesity and risk of cancer in postmenopausal Korean women. *Journal of clinical oncology : official journal of the American Society of Clinical Oncology* **26**, 3395-3402 (2008).
13. Kabat, G.C., Kim, M., Hunt, J.R., Chlebowski, R.T. & Rohan, T.E. Body mass index and waist circumference in relation to lung cancer risk in the Women's Health Initiative. *American journal of epidemiology* **168**, 158-169 (2008).
14. Jee, S.H. et al. Body mass index and cancer risk in Korean men and women. *Int J Cancer* **123**, 1892-1896 (2008).
15. Kabat, G.C., Miller, A.B. & Rohan, T.E. Body mass index and lung cancer risk in women. *Epidemiology* **18**, 607-612 (2007).
16. Reeves, G.K. et al. Cancer incidence and mortality in relation to body mass index in the Million Women Study: cohort study. *Bmj* **335**, 1134 (2007).
17. Samanic, C., Chow, W.H., Gridley, G., Jarvholm, B. & Fraumeni, J.F., Jr. Relation of body mass index to cancer risk in 362,552 Swedish men. *Cancer causes & control : CCC* **17**, 901-909 (2006).
18. Lukanova, A. et al. Body mass index and cancer: results from the Northern Sweden Health and Disease Cohort. *Int J Cancer* **118**, 458-466 (2006).
19. Batty, G.D. et al. Obesity and overweight in relation to organ-specific cancer mortality in London (UK): findings from the original Whitehall study. *International journal of obesity* **29**, 1267-1274 (2005).
20. Kuriyama, S. et al. Obesity and risk of cancer in Japan. *Int J Cancer* **113**, 148-157 (2005).

21. Rapp, K. et al. Obesity and incidence of cancer: a large cohort study of over 145,000 adults in Austria. *British journal of cancer* **93**, 1062-1067 (2005).
22. Oh, S.W., Yoon, Y.S. & Shin, S.A. Effects of excess weight on cancer incidences depending on cancer sites and histologic findings among men: Korea National Health Insurance Corporation Study. *Journal of clinical oncology : official journal of the American Society of Clinical Oncology* **23**, 4742-4754 (2005).
23. Liu, E., Wang, X., Yuan, J. & Gao, Y. Association of body mass index with risk of lung cancer: Evidence from a middle -aged male cohort in Shanghai, China. *Chinese J Clin Oncol* **2**, 90-95 (2004).
24. Calle, E.E., Rodriguez, C., Walker-Thurmond, K. & Thun, M.J. Overweight, obesity, and mortality from cancer in a prospectively studied cohort of U.S. adults. *The New England journal of medicine* **348**, 1625-1638 (2003).
25. Olson, J.E. et al. Differential association of body mass index and fat distribution with three major histologic types of lung cancer: evidence from a cohort of older women. *American journal of epidemiology* **156**, 606-615 (2002).
26. Drinkard, C.R. et al. Association of body mass index and body fat distribution with risk of lung cancer in older women. *American journal of epidemiology* **142**, 600-607 (1995).
27. Kark, J.D., Yaari, S., Rasooly, I. & Goldbourt, U. Are lean smokers at increased risk of lung cancer? The Israel Civil Servant Cancer Study. *Archives of internal medicine* **155**, 2409-2416 (1995).
28. Chyou, P.H., Nomura, A.M. & Stemmermann, G.N. A prospective study of weight, body mass index and other anthropometric measurements in relation to site-specific cancers. *Int J Cancer* **57**, 313-317 (1994).
29. Knekt, P. et al. Leanness and lung-cancer risk. *Int J Cancer* **49**, 208-213 (1991).
